# Characterization of Invasiveness, Thermotolerance and Light Requirement of Nine Invasive Species in China

**DOI:** 10.3390/plants12051192

**Published:** 2023-03-06

**Authors:** Arvind Bhatt, Xingxing Chen, Marcelo F. Pompelli, Aftab Jamal, Roberto Mancinelli, Emanuele Radicetti

**Affiliations:** 1 Lushan Botanical Garden, Chinese Academy of Sciences, Jiujiang 100101, China; 2 Facultad de Ciencias Agrícolas, Universidad de Córdoba, Montería 230002, Colombia; 3 Department of Soil and Environmental Sciences, Faculty of Crop Production Sciences, The University of Agriculture, Peshawar 25130, Pakistan; 4 Department of Agricultural and Forestry Sciences (DAFNE), University of Tuscia, 01011 Viterbo, Italy; 5 Department of Chemical, Pharmaceutical and Agricultural Sciences (DOCPAS), University of Ferrara, 44121 Ferrara, Italy

**Keywords:** invasiveness, mean germination time, relative light germination, seed shape, seed germination, synchrony

## Abstract

Understanding responsible functional traits for promoting plant invasiveness could be important to aid in the development of adequate management strategies for invasive species. Seed traits play an important role in the plant life cycle by affecting dispersal ability, formation of the soil seed bank, type and level of dormancy, germination, survival and/or competitive ability. We assessed seed traits and germination strategies of nine invasive species under five temperature regimes and light/dark treatments. Our results showed a considerable level of interspecific variation in germination percentage among the tested species. Both cooler (5/10 °C) and warmer (35/40 °C) temperatures tended to inhibit germination. All study species were considered small-seeded, and seed size did not affect germination in the light. Yet, a slightly negative correlation was found between germination in the dark and seed dimensions. We classified the species into three categories according to their germination strategies: (i) risk-avoiders, mostly displaying dormant seeds with low G%; (ii) risk-takers, reaching a high G% in a broad range of temperatures; (iii) intermediate species, showing moderate G% values, which could be enhanced in specific temperature regimes. Variability in germination requirements could be important to explain species coexistence and invasion ability of plants to colonize different ecosystems.

## 1. Introduction

Invasion by exotic plant species is recognized as a significant component of human-driven global environmental change, causing severe threats to biodiversity, ecosystem services, environmental quality, and human health [1,2,3,4]. Plant invasions cause huge economic losses due to their negative impact on agriculture, horticulture and natural ecosystems [5,6]. Invasive species usually display several features linked to their invasion success, such as (i) the ability to activate fast growth rates, (ii) high reproductive rates, (iii) greater dispersal capacity, and (iv) high adaptability to a broad range of environmental conditions [7,8,9]. Such characteristics have been found to be responsible for altering and reducing the native species’ composition/diversity by out-competing them [10], which ultimately leads to changes in ecosystem structure and function [11].

The presence of seed dormancy and the timing of dormancy relief may enable a unique ability of plants to survive under different environmental conditions by favoring seedling establishment in a suitable season [12]. Therefore, germination is one of the first stages of the plant life cycle, playing an important role in species survival, colonization, and distribution [13,14]. Understanding the germination strategies of invasive species could be important for developing adequate management strategies by identifying the factors responsible for inhibiting or stimulating their germination process [15,16,17]. Invasive species have been reported to show fast germination rates, reaching higher germination percentages under a wide range of environmental conditions [17,18,19,20,21]. However, germination strategies may vary among species, being affected by evolutionary history, life-history traits, and environmental conditions [22]. The study of germination strategies of different invasive species could be useful for understanding the ecological and evolutionary mechanisms of invasion.

In this context, light and temperature are the main environmental factors that control the germination process in space and time [12,23,24]. For example, light-dependent (positive photoblastic) seeds will only germinate if they remain upon or near the soil surface [25]. Temperature affects germination by regulating enzyme activities that promote/inhibit hormone synthesis and thus affect embryo growth [26]. Additionally, seed physical traits such as seed size, shape, color and structure may be a proxy to predict germination behavior, dormancy type and other seed functions, including dispersal mode [27]. Seed traits also play an important role in species invasiveness, as they may drive seed dispersal, germination timing and the ability to cope with environmental stress and disturbance [28]. Interspecific variations in seed traits have been linked to the variability in dispersal ability, the formation of soil seed banks, type and level of dormancy, germination, survival and/or competitive ability [29,30,31,32].

In the present study, we examined the overall differences in seed physical traits and germination strategies among nine widespread invasive species in Chinese landscapes. Specifically, we assessed: (i) a general description of seed traits (including seed color, dispersal mode, fresh weight, water absorption and seed shape; (ii) the effects of environmental factors (such as temperature and light) on germination parameters; (iii) correlations among variables. We hypothesized that different invasive species would exhibit differences in seed traits and germination strategies, and such heterogeneity could be important for their coexistence in similar environmental conditions. Regeneration from seeds may play an important role in determining species invasiveness, and understanding the role of seed traits could be useful for developing management strategies to halt the spread of invasive plants across different ecosystems worldwide.

## 2. Results

### 2.1. Species Characterization and Seed Physical Traits

Seven of the study species are annual plants (i.e., Abutilon theophrasti, *Geranium carolinianum*, *Lepidium virginicum*, *Plantago virginica*, *Solanum americanum*, *Veronica arvensis*, and Veronica persica), while Oenothera coronifera and *Phytolacca americana* display biennial and perennial habits, respectively (Supplementary Table S1). Most species occur in open areas (*A. theophrasti*, *O. coronifera*, *S. americanum*, and *V. persica*) and streamside (*G. carolinianum*, *L. virginicum*, *P. virginica*, and *V. arvensis*), but *P. americana* is found in forest habitats. Regarding invasion level, *P. americana* has been classified as level 1: severely invasive species. Other three species have been classified as level 2: highly invasive species (*G. carolinianum*, *L. virginicum*, and *V. persica*) and three more as level 3: locally invasive species (*A. theophrasti*, *P. virginica*, and *S. americanum*). The two remaining species (*O. coronifera* and *V. arvensis*) were classified as level 4: general invasive species (Supplementary Table S1).

The seed color was mostly brown, ranging from light brown (*P. virginica*) to dark brown (*O. coronifera* and *S. americanum*) and reddish brown (*L. virginicum*). Only *P. americana* seeds were black-colored (Table 1). Dispersal mode is predominantly mediated by animals—mostly endozoochory—but wind dispersal can also be found in *L. virginicum* and *V. persica* seeds. Seeds of *A. theophrasti* seem to show human-mediated dispersal—spread as a contaminant in grain and oilseeds, as registered in the literature (Table 1). In the field, we observed gravity (barochory) and water (hydrochory) as the main dispersal modes regarding seeds of *V. arvensis*.

**Figure 1:**
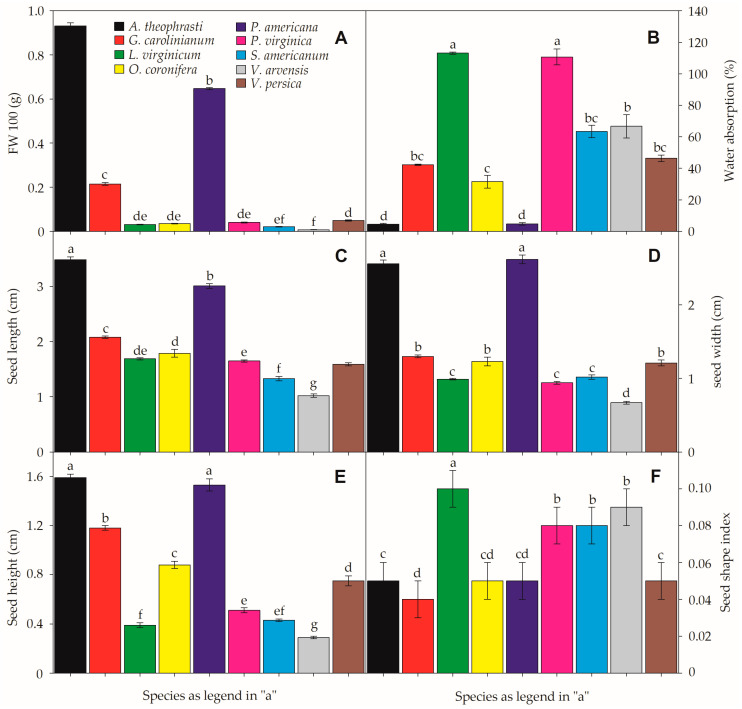
Seed functional traits of the nine invasive species. Fresh weight (**A**), water absorption (**B**), seed length (**C**) seed width (**D**), seed heighjt (**E**), and seed shape index (**F**). Small-case letters denote significant differences between species in each feature. All data represent the means (± SE). α = 0.01. For more details on the study species, please check Table 1 and Supplementary Table S1.

Four species had seed shape index values close to 0.1 (*L. virginicum*, *P. virginica*, *S. americanum*, and *V. arvensis*), indicating the presence of elongated or disc-shaped seeds. Their morphological classification ranged from ovate-oblong to narrowly ovoid and discoid seeds (Table 1). Although both *Veronica* species exhibited oblong-shaped seeds, *V. arvensis* tended to be more flattened/elongated (shape index = 0.09; Figure 1). All other study species had shape index values ≤0.05 (Figure 1), tending to show rounder seeds, including reniform-shaped (*A. theophrasti* and *P. americana*) and obovate-conic or prismatic seeds, respectively, in *G. carolinianum* and *O. coronifera* (Table 1).

Seed mass for 100 seeds ranged from 6.67 ± 1.33 (*V. arvensis*) to 930.671 ± 13.92 mg (*A. theophrasti*). Water absorption after 24 h ranged from 4.56% (*A. theophrasti*) to 113.10% (*L. virginicum*) (Figure 1). Thus, the fresh weight of seeds after imbibition significantly increased in most species except *A. theophrasti* (*p* = 0.28), *P. americana* (*p* = 0.15), and *V. arvensis* (*p* = 0.10), where the imbibition rate was null or very low. Similarly, seed length (SL) ranged from 1.02 ± 0.03 (*V. arvensis*) to 3.49 ± 0.05 mm (*A. theophrasti*). The same tendency was verified to seed weight (SW), ranging from 0.67 ± 0.02 (*V. arvensis*) to 2.56 ± 0.05 mm (*A. theophrasti*) and seed height (SH), which ranged from 0.29 ± 0.01 to 1.59 ± 0.03 mm respectively in the same species (Figure 1).

### 2.2. Role of Temperature on Germination

**Figure 2:**
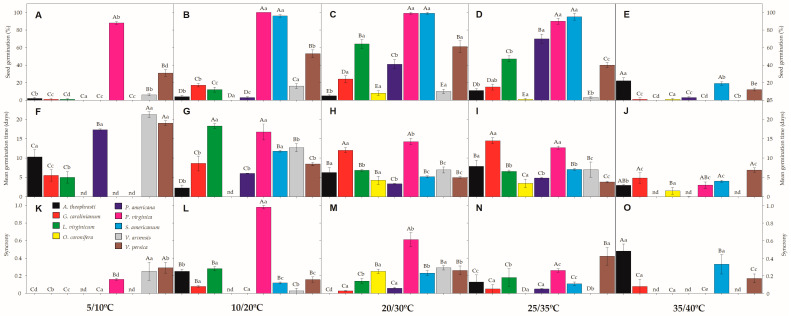
Germination (**A**–**E**), mean germination time (**F**–**J**), and synchrony (**K**–**O**) of the nine invasive species germinated in five different temperature regimes (5/10 °C; 10/20 °C; 20/30 °C, 25/35 °C, and 35/40 °C) in the light (12/12 h) photoperiod. Upper-case letters denote significant differences between species in each temperature incubation, and lower-case letters denote significant differences between temperature incubation within each species. nd denotes not determined due limit of the methodology. All values presented are the mean ± SE; α = 0.01. Color legend in K chart.

Under both extreme temperatures—either the coolest (5/10 °C) and the warmest (35/40 °C) tested conditions—germination percentage (G%) tended to be very low for all study species, except *P. virginica*, which showed 88% of germination in the coolest condition (Figure 2). However, seeds of four species had low G% under all tested temperatures (*A. theophrasti*, *G. carolinianum, O. coronifera*, and *V. arvensis*), barely reaching values from 8 to 24%. Most of such seeds remaining ungerminated by the end of the trials were found to keep intact/viable embryos (see Table 2). The highest G% was found (always in the light treatments) for *P. virginica* and *S. americanum* seeds, varying from 90 to 100% at the temperature regimes of 10/20 °C, 20/30 °C and 25/35 °C. The other two species (*L. virginicum* and *V. persica*) reached their highest G% when incubated at 20/30 °C, varying from 61 to 64% of germination (Figure 2). The temperature regime of 25/35 °C was best for the germination of *P. americana* seeds (70%).

Regarding germination times, MGT values were delayed in the coolest condition, lasting around 17 days for germination of *P. virginica* seeds (Figure 2). Likewise, *V. persica* seeds lasted around 19 days to germinate at 5/10 °C, while all other species had low G% (<20%) under this condition (as described above). MGT varied from 3 to 11 days to complete germination of *P. virginica*, *S. americanum* and *V. persica* seeds in the other temperature conditions (i.e., 10/20 °C, 20/30 °C, 25/35 °C). Seeds of *L. virginicum* took from 6 to 7 days to germinate, on average, under 20/30 °C and 25/35 °C, while *P. americana* seeds lasted twice the time (from 12 to 14 days) at the same temperature regimes (Figure 2). At 35/40 °C, MGT tended to be faster (e.g., 3 to 4 days), but followed by low G% (see above). Synchrony had values close to zero (Figure 2), showing that germination patterns are mostly scattered through time. The exception was found in *P. virginica* seeds, which reached 0.98 at 10/20 °C, showing a great synchronization of seeds germinating at the same time (Figure 2).

### 2.3. Light Requirements for Germination

All the studied species had small-seeded and mostly light-dependent (positive photoblastic) responses in at least one of the tested temperatures (Table 3). Nevertheless, light-requirement patterns were strongly linked to temperature regimes in three study species (*A. theophrasti*, *G. carolinianum*, *L. virginicum*). In spite of the low G% values, *A. theophrasti* and *G. carolinianum* seeds shifted from a negative to a positive photoblastic (often non-photoblastic) response according to the temperature regime. For instance, *A. theophrasti* seeds presented a negative response at 20/30 °C and 25/35 °C (reaching 28% of germination in the dark, relative light germination index (RLG) ~0.3, but had a positive photoblastic response under the temperature of 35/40 °C (22% in the light, RLG = 1, see Table 3). Seeds of *G. carolinianum* were classified as non-photoblastic under 10/20 °C (G% from 17 to 22%), negative photoblastic at 20/30 °C (reaching 37% of germination in the dark, RLG = 0.39) and positive photoblastic at 25/35 °C (15% in the light, RLG = 1, see Table 3). For *L. virginicum*, germination was negative photoblastic at 20/30 °C (45% in the dark, RLG = 0.21) and positive photoblastic at 20/30 °C and 25/35 °C (RLG from 0.76 to 1, Table 3). In contrast, all other species mainly had positive photoblastic responses under all tested temperatures where germination occurred. Seeds of *P. americana*, for instance, have shown a strong positive photoblastism with RLG values of 1 (germination occurring exclusively in the light; null G% in the dark) at the temperatures of 20/30 °C and 25/35 °C. Similarly, *P. virginica* seeds displayed positive photoblastic germination in all conditions where germination could be observed, with RLG values ranging from 0.8 to 1 (Table 3). *S. americanum* also had RLG values ranging from 0.8 to 1, with the exception of the temperature of 10/20 °C, where G% in the dark reached 69% (RLG = 0.58, Table 3). *V. arvensis* seeds tended to show a positive photoblastism when incubated at 10/20 °C and 20/30 °C, despite the low G% values. The germination response of *V. persica* was predominantly positive photoblastic at all temperature regimes, except the coolest condition (5/10 °C), where germination varied from 23% to 31% in dark and light respectively (thus non-photoblastic, RLG = 0.57, Table 3).

### 2.4. Correlation among Variables

Canonic correlation estimated in seeds germinated in light (CC1; Table 4) showed a non-significant correlation for any germination features, except for the thermotolerance index (0.9927). For seed germination in the dark, the canonic correlation (CC2), there was a significant relationship with some germination parameters (*i* = 0.7926; *p* = 2.67 × 10^−5^, see Table 4). For example, CC2 and seed fresh weight after 24 h (*r* = −0.1695) and seed width (*r* = −0.192) were direct but weakly significant. A moderate and inverse canonical correlation was found between CC2 and initial fresh weight (*r* = 0.1808), seed length (*r* = 0.3496), and seed shape (*r* = 0.2787). The canonical correlation between CC2 and RLG was strong and inversely proportional (*r* = 0.8353, see Table 4).

Pearson’s correlation showed a moderate and positive relationship between G% (in light) and synchrony (*r* = 0.118; *p* = 0.033; Supplementary Data File) and a weak but significant correlation between G% and MGT (*r* = 0.182; *p* = 9.64 × 10^−4^). However, the correlation between MGT and SYN was not significant (*p* = 0.431). Seed germination in light also had a weak but positive correlation with germination in the dark (*r* = 0.426; *p* = 5.32 × 10^−16^) and RLG index (*r* = 0.152; *p* = 0.014). Likewise, there was a weak-positive correlation of G% with seed length (*r* = 0.105; *p* = 0.045) and seed shape index (*r* = 0.290; *p* = 8.27 × 10^−8^). Contrastingly, G% was negatively correlated (*r* = −0.171; *p* = 2.02 × 10^−3^) to water absorption after 24 h (Supplementary Data File).

Germination in the dark had a weak and negative correlation with the initial fresh weight (*r* = −0.193; *p* = 4.71 × 10^−3^) and seed dimensions: seed length (*r* = −0.172; *p* = 0.002), width (*r* = −0.208; *p* = 1.46 × 10^−4^), and height (*r* = −0.154; *p* = 0.005). Also, G% in the dark had a strongly negative correlation with the RLG index (*r* = −0.570; *p* = 8.05 × 10^−24^), but it was not correlated to seed shape (*p* = 0.743). Because only the final germination percentage was computed in the dark treatments, neither MGT nor SYN could be calculated. In seeds germinated in light, synchrony was weakly and positively correlated with water absorption (*r* = 0.217; *p* = 2.17 × 10^−5^) but negatively correlated to the initial fresh weight (*r* =−0.201; *p* = 2.81 × 10^−4^), as well as to seed dimensions (length, width, height; *r* values around −0.3, see Supplementary Data File) and to RLG (*r* = −0.124; *p* = 0.04). Fresh seed weight was mostly positively correlated with all seed dimensions and water absorption but negatively correlated to the seed shape index (*r* = −0.35). Water absorption was negatively correlated to seed length and seed shape index (*r* = −0.296 and −0.403, respectively). In a practical sense, seed length, width, and height were strongly correlated to each other (*r* values around 0.9), and all seed dimensions were negatively related to seed shape (Supplementary Data File).

### 2.5. Dendrogram

**Figure 3:**
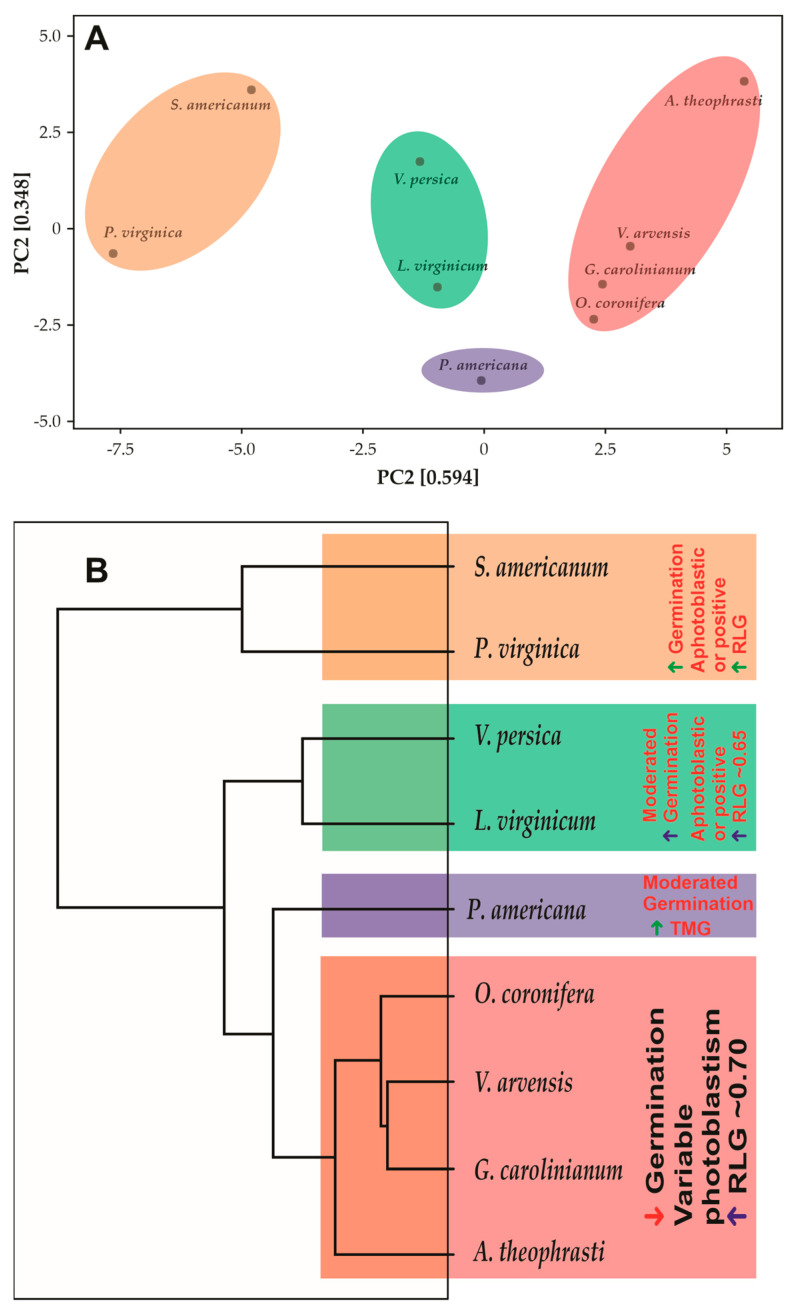
(**A**). Principal component analysis showing the 4 distinct groups in a cartesian plan (PC1 and PC2). (**B**). Dendrogram showing the diversity of species, showing branches less differentiated (top of figure) and highly differentiated branched (on the base).

The PCA resulted from a multifactorial analysis of all evaluated characteristics showed that the species described in this study form well-defined 4 groups, with a PC1 + PC2 summing 0.942, which means that the PCA represents 94.2% of the variations that may occur within analyses (Figure 3). The different groups do not share with other, being totally clear and concentric. Reading the dendrogram from top to bottom, the first group is formed by the species *S. americanum* and *P. virginica*. The second group is formed by the species *V. persica* and *L. virginicum*. The species *O. coronifera*, *V. arvensis*, *G. carolinianum* and *A. theophrasti* form a third group, while *P. americana* appears to be completely isolated from the other species, forming a group completely distant from the others by at least 55.5% similarity. The group formed by the species *S. americanum* and *P. virginica* differs from the others by presenting high synchrony in the germination of the seeds incubated between 10–35 °C, by presenting an ovoid or discoid shape, by presenting high thermotolerance and high germination in the dark in the seeds incubated at 10/20 °C. The group formed by the species *O. coronifera*, *V. arvensis*, *G. carolinianum* and *A. theophrasti* present similar characteristics, such as high seed viability after germination in the dark, photoblastism at 20/30 °C, where the species *G. carolinianum* and *A. theophrasti* show negative photoblastism, while *O. coronifera* and *V. arvensis* showed positive photoblastism. However, the predominant photoblastism in this group is positive, with values approaching 1, which means photoblastism-positive in this study. Also, it is common in this group that seeds have higher seed lengths. The species *V. persica* and *L. virginicum* share the second-highest seed absorption (79.8%), followed by the group formed by the species *S. americanum* and *P. virginica* (87%). Furthermore, the group formed by the species *V. persica* and *L. virginicum* share the highest synchrony in germination in seeds incubated at 20/30 °C (0.42) and the highest thermotolerance among the studied species. On the other hand, the group formed by the species *S. americanum* and *P. virginica* share the highest seed absorption (87%), the highest seed germination in the dark (at 10/20 °C—43%), the highest synchrony in seeds incubated at 25/35 °C (0.19), and the highest germination in light at 20/30 °C (99%).

## 3. Discussion

Although the study species frequently shared common physical/morphological traits such as small seeds, we have found contrasting germination strategies among them. For instance, four species (*A. theophrasti*, *G. carolinianum*, *O. coronifera*, and *V. arvensis*) displayed low G% values in all tested temperatures but kept high viability of seeds, which indicates the presence of some type of dormancy [12]. The other two species (*P. virginica* and *S. americanum*) had high G% in a broad range of temperatures. Previous studies have already reported that *A. theophrasti* seeds are physically dormant due to the presence of an impermeable seed coat [39], as confirmed by our water imbibition tests. Seeds of *G. carolinianum* might also display water-impermeable seed coats [40], although our imbibition tests detected an average of 40% of water absorption in the samples (Figure 1). Different species of *Oenothera* seem to show a physiological type of dormancy [41], as well as described to *Veronica* spp. in the literature [42]. The identification of species traits related to invasiveness is relevant for predicting which species might become invasive [43,44]. Moreover, dormancy and germination are important traits and play a vital role in the establishment of invasive species [17].

The presence of dormancy helps in the formation of soil seed banks, also assisting in optimizing MGT [28,45]. Therefore, species bearing dormant seeds can be considered species that avoid taking risks to germinate. Physiological dormancy is caused by embryo mechanisms requiring specific environmental cues that allow germination to occur. A species could thus show fast germination when seasonal events are favorable, while germination can be delayed (scattered in time) when the environmental conditions are unfavorable for seed germination and seedling establishment [24]. However, we observed a considerable level of interspecific variation in germination percentage among the tested species, and this variability could be linked to the presence of different types and levels of dormancy among them. Such variation can be observed even within the germination patterns of a single species.

Hicks et al. [46] reported that *P. virginica* seeds show physiological dormancy in their native environments [46], in disagreement with this study, where this species registered G% ranging from 88% to 100% in most of the tested temperatures. *P. virginica* also had a high synchrony of germination, depending on the temperature regime (e.g., 10/20 °C), with MGT ranging from 3 to 6 days (except in the coolest temperature, see Figure 2). The other study species, which promptly germinated in a wide range of temperatures (*S. americanum*), had MGT values varying from 5 to 11 days, thus also being considered as a risk-taking strategist. Germination ability in a wide range of temperatures may reflect a species’ capacity to occupy a broad regeneration niche in a time-thermal-spatial way, which seems to be a common strategy among invasive species [47,48]. Although *P. virginica* and *S. americanum* were classified as level 3 of invasion (locally invasive species), their broad germination capacity indicates a potential to expand to non-invaded areas, tending to become more problematic in the near future. Similar results have been suggested by Ozaslan et al. [17] in seeds of *Physalis* spp. (Solanaceae) infesting arid and semi-arid regions of Turkey.

In addition to the dormant (risk-avoiding) and germinant (risk-taking) strategies, three study species had intermediate levels of dormancy, mostly depending on specific temperature regimes to germinate. It is the case of *P. americana*, the most severely invasive study species, which has been reported to show physically dormant seeds [49], but here we registered up to 70% of germination at 25/35 °C (Figure 2). Water-impermeable seeds have demonstrated dormancy alleviation under alternating temperature regimes in many species (mostly legumes) worldwide, including the problematic *Leucaena leucocephala* [50]. Likewise, *L. virginicum* (up to 64%) and *V. persica* (up to 61%) seeds reached their maximal values of G% under the temperature regime of 20/30 °C. Such moderate seed germination indicates that the fraction of seeds remaining ungerminated (but viable) probably had physiological dormancy or at least a slower germination rate. This variation in dormancy and germination among species and populations could be driven by geographical variation in environmental factors (i.e., temperature and precipitation) as reported for various invasive species [51,52,53].

Our results suggest that although the invasive species may grow in similar environmental conditions, they do not show common germination strategies; each species seems to display its own germination requirements. Temperature is widely known as one of the most important environmental factors regulating seed dormancy and germination [12]. Previous studies reported that the optimal temperature requirement for germination is species-specific [54,55]. Such patterns have been described for different ecosystems worldwide, including Arabian deserts [56], Mediterranean systems [57] and tropical forests [58]. A variability in germination requirements could contribute to species coexistence by spreading recruitment in time and space, also reducing competition for resources [59]. However, each species (or population) has a proper temperature range (lower and upper limits) for germination [60], which can be used to understand the thermal tolerance of the regeneration niche [61]. The characterization of such temperature thresholds for germination can define the limits of the thermal environment that a species will tolerate [62,63]. These temperatures match the germination timing to favorable conditions for seedling growth and establishment [64]. We found that temperature had a marked effect on the germination of the nine invasive species.

Previous reports have stated that the lower temperature limit for germination is related to ecological adaptation, while the upper limit is caused by physiological constraints [65]. A nearly null proportion of seeds were able to germinate at the coolest temperature regime, except for *P. virginica* (88%) and *V. persica* (31%). Hence, *avoiding germination in winter (December to February), when the temperature is around* 5–10 °C, *could be a common adaptation strategy in the collection areas. If seeds germinated during this time, their chances of successful seedling recruitment would be drastically reduced due to cold and frost.* Similarly, the warmest treatment (35/40 °C) also severely inhibited the germination of all study species, indicating their sensitivity to high-temperature conditions. However, seed viability remained high after incubation under these extreme temperatures, indicating that seeds might promote a dormancy state and remain viable in the soil seed bank until they experience appropriate temperature conditions for germination. High and low temperatures may play important roles in the induction of secondary seed dormancy [66,67]. Moreover, high temperature has been related to higher levels of endogenous abscisic acid (ABA), which up-regulate ABA biosynthesis genes and down-regulate catabolism genes, thus inhibiting germination [68,69].

Light requirements for germination also varied with temperature. For example, *S. americanum* seeds had a relatively high G% (up to 69%) in the dark at 10/20 °C, tending to follow a non-photoblastic pattern (RLG = 0.58), but seeds showed a higher dependence of light for germination (RLG values close to 1) with increasing temperatures. Seeds of *L. virginicum* even achieved significantly greater germination in the dark (G% = 45%) as compared to light (12%) at 10/20 °C, but also shifted to a positive photoblastism with the increasing temperatures at 20/30 °C and 25/35 °C. These results indicate that interactions of temperature and light drive the capacity of seeds to germinate, and therefore seeds may require light at a certain temperature regime but not at others [70]. The seeds of some species remain ungerminated at dark conditions under low and mild temperatures, but their light requirement can be reduced at warmer conditions, as reported to Velloziaceae species in rocky outcrops [71]. In other cases, higher temperatures impose the positive photoblastic response, with the absence of germination in the light at lower temperatures, but light requirement becomes progressively manifested when the temperature exceeds a certain threshold [72].

Seed size had little to no influence on G% in the light treatments. For instance, *P. virginica* and *S. americanum*, which showed the highest G% values, had a seed length from 1.33 to 1.65 mm—similar to other species (*L. virginicum* and *V. persica*) that reached intermediate germination. Regarding seed shape, seeds of *L. virginicum* had the highest values (0.1, tending to show elongated/flattened seeds), followed by *P. virginica*, *S. americanum* and *V. arvensis*. However, G% in the light had a negative correlation with water absorption and a positive correlation with the seed shape index. Higher germination rates have been linked to a higher percentage of water in the seeds, which leads to less negative osmotic potentials and high respiration rates in seeds [73]. Chidananda et al. [74] also described that the respiration rate increased with seed moisture content and that seed germination increased linearly with seed respiration. Increasing temperature leads to an increase in the same proportion in the respiratory rate [73,75], but this only becomes possible if there is enough water, up to a temperature threshold, as heating generates latent heat dissipation from vaporization.

The multifactorial analysis and principal component analysis allowed us to group the species into four large groups: (i) group showing a high germination rate (higher than 85%), photoblastism ranging from non-photoblastic to positive, and RLG higher than 0.85; (ii) group predominantly composed of species with low germination rate (~8.5%), very variable photoblastism depending on the incubation temperature, and RLG ~0.70; (iii) group of species showing moderate germination (~36%), photoblastism ranging from non-photoblastic to positive, and RLG higher than 0.65. The species *P. americana* did not permit any type of grouping, maybe for presenting intermediary germination only at temperatures between 20–35 °C and a high TMG. Also, *P. americana* is the only species that inhabits forests, and this species shows level 1 invasion, i.e., it is a severely invasive species. The grouping classification to higher G and RLG, plus non-photoblastic to positive photoblastism, was also described by 11 succulent species from the southern Chihuahuan Desert, Mexico [76]. The same pattern was described to *Discocactus* sp. grown in Caatinga, Brazil, a Savanna-like ecosystem [77,78,79]. Meiado [79] describes that plants with these characteristic commonly present an invasive habitat of high propagation and difficult to control, while Flores et al. [38] describes that lighter seeds tend to have higher RLG, a fact that is in agreement with this study. Cheib and Garcia [80] described that in the presence of light, lower seeds of *Arthrocereus* sp. show low germination percentages at 10, 15 and 35 °C, a pattern similar to that described in this work for the species *A. theophrasti*, *O. coronifera*, *G. carolinianum*, and *V. arvensis*. In accord with these authors this behavior may represent an adaptive mechanism during seasons when environmental conditions in open rocky fields are not favorable for seedling survival. Intermediate size seeds, with moderate germination rate and high RLG were also described by Rojas-Aréchiga, et al. [81] in species belonging to tribe Cacteae, in Mexico. Shaikh, et al. [82] reported strong positive photoblastism in seeds of a Pakistani *E. ciliaris*. Furthermore, these authors recorded high-light germination at higher temperatures (25/35 °C), as shown in this study for *L. virginicum* and *V. persica*.

It is known that larger seeds might be independent of light to germinate [37]. *P. americana* seeds were revealed to be strongly positive photoblastic, while *A. theophrasti* seeds had low G% both in light and dark. The weak (and inverse) correlations between germination in darkness (CC2) and fresh seed weight also denote that small seeds might often germinate in the dark treatments in a few temperature conditions, as we registered to *G. carolinianum*, *L. virginicum*, and *S. americanum* (seed length from 1.33 to 2.08 mm). Conversely, elongated (or flattened) seeds could also have greater germination in the dark, as corroborated by the positive correlation between CC2 and seed shape. Funes et al. [83] demonstrated that seed size and shape are important determinants of persistence in the soil for 71 herbaceous species from a montane grassland in Argentina, where small and compact seeds tend to persist (ungerminated) in the soils for longer periods of time. This pattern contradicts Leishman and Westoby [84], who analyzed the relationship between seed size and shape and persistence in the soil for 101 Australian species from a range of habitats and found that seed size and shape were not related to persistence in the soil. Such relationships might still remain unclear regarding invasive species in Chinese landscapes. A proportion of seeds that eventually germinate in the dark at some mild temperatures seems to indicate that buried seeds would be less affected by extreme climatic variation, as well as protected from frugivorous [85] and fast decomposition. Notwithstanding, those seeds would be running the risk of not effectively generating seedlings due to their smaller seed size and fewer embryo reserves, which may not be enough to reach the soil surface before showing positive net photosynthesis [86]. Hence, larger seeds have more probability of surviving and generating new plants even though they are buried [87]. Flores and Briones [88] showed that RLG decreased as the seed mass increased in a Mexican desert. Rojas–Aréchiga et al. [81], in contrast, did not find any evidence between the seed size and photoblastic responses, suggesting that photoblastism was not of adaptive origin. Seed responses to light are very plastic and might change whether the germinative conditions are naturally or artificially altered.

## 4. Materials and Methods

### 4.1. Seed Collection

**Figure 4:**
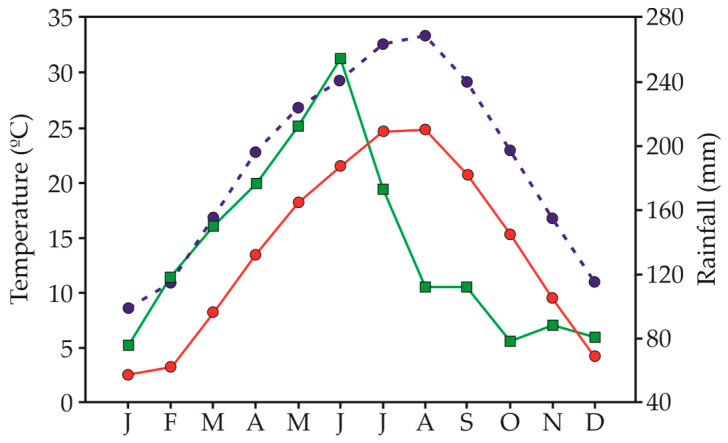
Minimum temperature (red circles), maximum temperature (blue circles) and precipitation (green squares) registered as the mean of the 5 years after the start of the experiment (2015–2019) in Guling, Jiangxi, China.

The seeds of 9 different invasive species were collected at the time of their natural dispersal to ensure seed maturity in 2022. Most of the study species are annuals and originated from America (Supplementary Table S1). Based on their severity impact, the selected invasive species have been categorized into 4 different levels [89] (Supplementary Table S1). For each species, seeds were collected from 25 to 30 randomly chosen plants to represent the genetic diversity of the population. After collection, all seeds were cleaned and immediately tested for germination within a week after collection. The climate in the collection areas (Jiujiang, China) follows monsoonal patterns of precipitation, with rainfall events scattered throughout the year but a peak of precipitation between May and June (Figure 4). Temperature varies greatly within the year, with July and August being the hottest and December to January being the coldest months.

### 4.2. Microscopy Methods

Seed dimensions (e.g., length, width, and height) were measured by using a Stereo Microscope (Nikon SMZ800N; Nikon Instruments Inc., Melville, NY, USA) coupled with a microscope camera IMG-SC600C (iMG Biotechnology Co., Ltd., Suzhou, Jiangsu, China). The seed dimensions were used to calculate the seed shape index (SS) as SS = $Variance\;\left(\frac{length}{length},\;\frac{width}{length},\;\frac{height}{length}\right)$ according to Thompson et al. [90]. This variance has a minimum value of zero in perfectly spherical diaspores and maximal values of about 0.3 in needle- or disc-shaped diaspores. A total of 15 seeds per species were examined, attaching them ventrally to filter paper using double-sided sticky tape. Seed color also was described using the Stereo Microscope described above. Seed mass was determined at the time of collection from 3 100-seed replicates per species, using an analytical balance (Sartorius Analytical Balance mod. ENTRIS224-1S, Bradford, MA, USA; accurate to 0.1 mg).

### 4.3. Water Imbibition

Seed permeability to water was assessed by recording the mass of 3 100-seed replicates before and after placing them in a 25 mL beaker containing 15 mL of deionized water for 24 h at room temperature (22 ± 2 °C). Water absorption was expressed as a percentage of change in mass [12]. Regression curves showed that in 24 h, all the seeds were completely turgid, i.e., point of being all seeds were submerged in water (data not shown).

### 4.4. Seed Germination

To determine the effect of temperature and light, seed germination was conducted in incubators (Kesheng incubators, Model DRX-800C-LED, Pequim, China) set at different alternate temperature regimes (5/10 °C, 10/20 °C, 20/30 °C, 25/35 °C, and 35/40 °C) in either 12-h light/12-h darkness (light treatment) and 24-h darkness (dark treatment). The incubators were fitted with cool-white fluorescent tubes (60 µmol photons m^−2^ s^−1^). The tested temperatures were chosen to stimulate the average temperature regimes in different months throughout the year (i.e., 5/10 °C—December to February, 10/20 °C—March to April and October to November, 20/30 °C—May, June and September, 25/35 °C—July and August) at the seed collecting area. In addition, a higher temperature regime (35/40 °C) was applied in order to investigate the ability of seeds to tolerate warmer conditions during germination as a consequence of the greenhouse effect and global climatic change [91].

Seeds were surface sterilized in 0.5% sodium hypochlorite for 1 min and subsequently washed thrice with deionized water to avoid fungus attack. Then, seeds were sown in 9 cm Petri dishes lined with 3 layers of disks of Whatman No. 1 filter paper, moistened with 10 mL of distilled water and placed in incubators. Darkness was achieved by wrapping the Petri dishes in 2 layers of aluminum foil. Four replicates of 25 seeds each were used for each treatment per species. The seeds were considered germinated with the emergence of the radicle by ≥2 mm through the external integument, as proposed by the International Seed Testing Association [92]. Germinated seeds were counted and removed daily for a 30-d period. However, seeds incubated in the dark were checked only at the end of the test. Thereafter, seed germination (G%), mean germination time (MGT), and synchrony (SYN) were computed using GerminaR [93]. At the end of the germination tests, all remaining ungerminated seeds from the light treatment were dissected under a Stereo Microscope to evaluate their embryo status and viabilities. Seeds bearing visibly intact and clear embryos were considered viable; turgid/damaged and brownish as dead.

Moreover, we classified germination dependence on light (photoblastism) considering 3 categories: positive, negative, and non-photoblastic seeds. We also calculated the relative light germination (RLG) index, as described by Milberg et al. [37] and Flores et al. [38], which can be determined as $RLG=\frac{GL}{GD+GL}$, where GL = germination percentage in light, and GD = germination percentage in darkness. RLG represents a range of values varying from 0 (germination only in darkness) to 1 (germination only in light). To access the temperature preference, we calculated the thermotolerance index as $TI=\frac{GL}{GL+VS+DS}$, where GL = germination percentage in light, VS = viable seeds but non-germinated, and DS = non-viable seeds or dead seeds.

### 4.5. Dendrograms

All analyzed features were used to make the dendrogram analysis. The main features used were thermotolerance and light requirement in seed germination. For the dendrogram construction, all analyzed features were used to draw the dendrogram, and the grouping was made taking into account the principal component analysis. Thus, all components were imputed in the Minitab^®^ 18.1 (Minitab LLC Inc., State College, PA, USA), where the similar or distal characteristics were analyzed using a dendrogram.

### 4.6. Data Analysis

The influence of incubation temperature on 3 dependent variables (germination percentage, mean germination time, and synchrony) was performed using GerminaR software [93]. All the data were analyzed by ANOVA, and means were compared using an SNK test (*p* < 0.05) by Statistic version 14.0 (StatSoft, Tulsa, OK, USA). Correlations among variables were assessed using Pearson correlations using Sigmaplot version 14.0 (Systat Software Inc., San Jose, CA, USA). All regression analysis was performed using Data Fit version 8.0.32 (Oakdale Engineering, Oakdale, PA, USA).

## 5. Conclusions

We found significant differences in seed physical and physiological (germination) traits among the studied species. Each species seemed to show specific temperature requirements to achieve the greatest germination. However, the extreme temperatures (5/10 °C and 35/40 °C) mainly inhibited germination. The light requirements for germination may also change according to temperature regimes. Additionally, seed size had no effect on germination in the light but had a slightly negative correlation with seed dimensions in the dark treatments.

In summary, based on their germination strategies to temperature, we categorized the studied species into three groups: (i) risk-avoiders: species that showed low G% (<30%) in all temperature regimes (*A. theophrasti*, *G. carolinianum*, *O. coronifera*, *V. arvensis*), mostly bearing dormant seeds; (ii) risk-takers: species that displayed a high G%, reaching 90% of germination in a broad range of temperatures (*P. virginica*, *S. americanum*); (iii) intermediate species: usually had moderate G% values, with a proportion of seeds remaining dormant, but germination could be enhanced in specific temperature regimes (*L. virginicum*, and *V. persica*). *P. americana* shares some results with the last group; however, the PCA showed that this species did not show any share with the other three groups. These results could contribute to a better understanding of the distribution of invasive species and their ability to spread in non-invaded areas and other ecosystems worldwide.

## Figures and Tables

**Table 1:** Seed morphology and dispersal mode of the nine invasive species. Traces indicate the absence of relevant literature about dispersal mode. For more details of the study species, such as family, collection date and site, habit, habitat, invasion level and origin, please check Table S1 (Supplementary Materials).

Species	Shape	Color	Dispersal Mode	Reference
*Abutilon theophrasti* Medik.	Reniform	Brown	Human-mediated dispersal	Follak et al. [33]
*Geranium carolinianum* L.	Long obovate-conic	Brown	Epizoochorous	Qi-He et al. [34]
*Lepidium virginicum* L.	Ovate-oblong	Reddish brown	Wind/Animal dispersed	Zhang et al. [22]
*Oenothera coronifera* Renner	Prismatic	Brown to dark brown	Endozoochoric	----
*Phytolacca americana* L.	Reniform-auricular	Black	Animal dispersed	Qi-He, et al. [34]
*Plantago virginica* L.	Ovoid to narrowly ovoid	Light brown	Endozoochoric	----
*Solanum americanum* Mill.	Discoid	Dark brown	Endozoochoric	Carlo [35]
*Veronica arvensis* L.	Oblong	Brown	Barochory/hydrochory	----
*Veronica persica* Poir.	Oblong	Brown	Endozoochoric	Weiner et al. [36]

**Table 2:** Germination percentage in the light treatments, percentage of seeds remaining viable and non-viable by the end of the trials, analyzed under a stereoscope, and thermotolerance index (TI) of the nine invasive species at five different temperature regimes. The thermotolerance index was calculated as described in Material and Methods.

Species	5/10 °C				10/20 °C				20/30 °C				25/35 °C				35/40 °C			
	Germ.	Viable	Non-Viable	TI	Germ.	Viable	Non-Viable	TI	Germ.	Viable	Non-Viable	TI	Germ	Viable	Non-Viable	TI	Germ	Viable	Non-Viable	TI
*A. theophrasti*	2.00	**96.00**	2.00	0.02	4.00	**93.00**	3.00	0.04	5.00	**93.00**	2.00	0.05	11.00	**86.00**	3.00	0.11	22.00	**75.00**	3.00	0.22
*G. carolinianum*	1.00	**97.00**	2.00	0.01	17.00	**81.00**	2.00	0.17	24.00	**74.00**	2.00	0.24	15.00	**83.00**	2.00	0.15	1.00	**97.00**	2.00	0.01
*L. virginicum*	1.00	**97.00**	2.00	0.01	12.00	**86.00**	2.00	0.12	**64.00**	34.00	2.00	**0.64**	47.00	**51.00**	2.00	0.47	0.00	**98.00**	2.00	0.00
*O. coronifera*	0.00	**97.00**	3.00	0.00	0.00	**98.00**	2.00	0.00	8.00	**90.00**	2.00	0.08	1.00	**97.00**	2.00	0.01	1.00	**96.00**	3.00	0.01
*P. americana*	0.00	**97.00**	3.00	0.00	3.00	**94.00**	3.00	0.03	41.00	**56.00**	3.00	0.41	**70.00**	29.00	1.00	**0.70**	3.00	**94.00**	3.00	0.03
*P. virginica*	**88.00**	11.00	1.00	**0.88**	**100.00**	0.00	0.00	**1.00**	**99.00**	0.00	1.00	**0.99**	**90.00**	9.00	1.00	**0.90**	0.00	**98.00**	3.00	0.00
*S. americanum*	0.00	**96.00**	4.00	0.00	**96.00**	2.00	2.00	**0.96**	**99.00**	0.00	1.00	**0.99**	**95.00**	3.00	2.00	**0.95**	19.00	**79.00**	2.00	0.19
*V. arvensis*	6.00	**92.00**	2.00	0.06	16.00	**83.00**	1.00	0.16	10.00	**88.00**	2.00	0.10	3.00	**95.00**	2.00	0.03	0.00	**97.00**	3.00	0.00
*V. persica*	31.00	**67.00**	2.00	0.31	**53.00**	45.00	2.00	0.53	**61.00**	36.00	3.00	**0.61**	40.00	**56.00**	4.00	0.40	12.00	**86.00**	2.00	0.12

**Table 3:** Germination in light and dark treatments of the nine invasive species at five different temperature regimes. Photoblastism (class) and relative light germination index (RLG) were calculated as proposed by Milberg et al. [37] and Flores et al. [38]. The *p*-value denotes the significance of germination in light and dark treatments at *p* ≤ 0.001 (***), *p* ≤ 0.01 (**), *p* ≤ 0.05 (*), or not significant (ns).

Species	Temperature	Germination (%)		Photoblastism (Class)	RLG	*p* Value	Significance
		Light	Dark				
*A. theophrasti*	5–10 °C	2.0 ± 1.2	2.0 ± 1.2	Non-photoblastic	0.50	1	ns
	10–20 °C	4.0 ± 1.6	6.0 ± 2.0	Non-photoblastic	0.40	0.47	ns
	20–30 °C	5.0 ± 1.0	11.0 ± 1.9	Negative	0.31	0.03	*
	25–35 °C	11.0 ± 1.9	28.0 ± 1.6	Negative	0.28	5.13 × 10^−4^	***
	35–40 °C	22.0 ± 3.5	0.0 ± 0.0	Positive	1.00	7.14 × 10^−4^	***
*G. carolinianum*	5–10 °C	1.0 ± 1.0	8.0 ± 2.3	Negative	0.11	0.03	*
	10–20 °C	17.0 ± 1.9	22.0 ± 1.2	Non-photoblastic	0.16	0.07	ns
	20–30 °C	24.0 ± 4.0	37.0 ± 1.9	Negative	0.39	0.03	*
	25–35 °C	15.0 ± 3.4	0.0 ± 0.0	Positive	1.00	4.61 × 10^−3^	**
	35–40 °C	1.0 ± 1.0	0.0 ± 0.0	Non-photoblastic	1.00	0.36	ns
*L. virginicum*	5–10 °C	1.0 ± 1.0	2.0 ± 1.2	Non-photoblastic	0.33	0.54	ns
	10–20 °C	12.0 ± 2.8	45.0 ± 5.5	Negative	0.21	1.78 × 10^−3^	**
	20–30 °C	64.0 ± 5.2	20.0 ± 1.6	Positive	0.76	1.87 × 10^−4^	***
	25–35 °C	47.0 ± 3.4	0.0 ± 0.0	Positive	1.00	9.16 × 10^−6^	***
	35–40 °C	0.0 ± 0.0	0.0 ± 0.0	Non-photoblastic	----	----	ns
*O. coronifera*	5–10 °C	0.0 ± 0.0	0.0 ± 0.0	Non-photoblastic	----	----	ns
	10–20 °C	0.0 ± 0.0	0.0 ± 0.0	Non-photoblastic	----	----	ns
	20–30 °C	8.0 ± 2.8	0.0 ± 0.0	Positive	1.00	0.03	*
	25–35 °C	1.0 ± 1.0	0.0 ± 0.0	Non-photoblastic	1.00	0.36	ns
	35–40 °C	1.0 ± 1.0	0.0 ± 0.0	Non-photoblastic	1.00	0.36	ns
*P. americana*	5–10 °C	0.0 ± 0.0	0.0 ± 0.0	Non-photoblastic	----	----	ns
	10–20 °C	3.0 ± 1.0	0.0 ± 0.0	Positive	1.00	0.02	*
	20–30 °C	41.0 ± 5.3	0.0 ± 0.0	Positive	1.00	2.35 × 10^−4^	***
	25–35 °C	70.0 ± 5.3	0.0 ± 0.0	Positive	1.00	1.15 × 10^−5^	***
	35–40 °C	3.0 ± 1.0	1.0 ± 1.0	Non-photoblastic	0.75	0.21	ns
*P. virginica*	5–10 °C	88.0 ± 1.6	6.0 ± 1.2	Positive	0.94	1.41 × 10^−8^	***
	10–20 °C	100.0 ± 0.0	17.0 ± 19.0	Positive	0.85	1.01 × 10^−8^	***
	20–30 °C	99.0 ± 1.0	22.0 ± 1.2	Positive	0.82	4.09 × 10^−9^	***
	25–35 °C	90.0 ± 3.5	0.0 ± 0.0	Positive	1.00	2.14 × 10^−7^	***
	35–40 °C	0.0 ± 0.0	0.0 ± 0.0	Non-photoblastic	----	----	ns
*S. americanum*	5–10 °C	0.0 ± 0.0	0.0 ± 0.0	Non-photoblastic	----	----	ns
	10–20 °C	96.0 ± 1.6	69.0 ± 2.5	Non-photoblastic?	0.58	1.05 × 10^−4^	***
	20–30 °C	99.0 ± 1.0	24.0 ± 1.6	Positive	0.80	1.85 × 10^−8^	***
	25–35 °C	95.0 ± 3.8	17.0 ± 2.5	Positive	0.85	2.51 × 10^−6^	***
	35–40 °C	19.0 ± 1.9	0.0 ± 0.0	Positive	1.00	6.05 × 10^−5^	***
*V. arvensis*	5–10 °C	6.0 ± 1.2	0.0 ± 0.0	Positive	1.00	0.002	**
	10–20 °C	16.0 ± 2.8	3.0 ± 1.0	Positive	0.84	0.005	**
	20–30 °C	10.0 ± 2.6	1.0 ± 1.0	Positive	0.91	0.02	*
	25–35 °C	3.0 ± 1.0	0.0 ± 0.0	Non-photoblastic	1.00	0.51	ns
	35–40 °C	0.0 ± 0.0	0.0 ± 0.0	Non-photoblastic	----	----	ns
*V. persica*	5–10 °C	31.0 ± 3.8	23.0 ± 3.4	Non-photoblastic	0.57	0.17	ns
	10–20 °C	53.0 ± 4.4	32.0 ± 1.6	Positive	0.62	0.004	**
	20–30 °C	61.0 ± 6.6	14.0 ± 1.2	Positive	0.81	4.21 × 10^−4^	***
	25–35 °C	40.0 ± 2.8	9.0 ± 1.0	Positive	0.82	4.80 × 10^−5^	***
	35–40 °C	12.0 ± 1.6	0.0 ± 0.0	Positive	1.00	3.25 × 10^−4^	***

**Table 4:** Coefficients of canonical correlations (CC) between seed germination in light (CC1) and dark (CC2) related to germination parameters, seed morphological traits, light requirement and thermotolerance in the nine invasive species.

Variables	CC1	CC2
Group 1		
Seed germination	1.0	−1.0
Group 2		
Mean germination time	0.0073	*
Synchrony	0.0108	*
Fresh weigth	0.0036	0.1808
Fresh weigth after 24 h	0.0283	−0.1695
Seed length	0.0622	0.3496
Seed width	0.0023	−0.1923
Seed height	−0.0442	−0.0311
Seed shape	−0.0106	0.2787
Relative light germination	0.0032	0.8353
Thermotolerance index	0.9927	−0.6240
*R* canonical	0.9955	0.7926
*p*-value	6.58 × 10^−8^	2.67 × 10^−5^

* mean germination time and synchrony were not analyzed in seed germination in darkness.

## Data Availability

All Supplementary Files were available free for download in https://1drv.ms/u/s!Ahh_7tJTDWeQscF3aO1E4mCcX5ETDQ?e=aHSh4B.

## Supplementary Materials

The following supporting information can be downloaded at: https://www.mdpi.com/article/10.3390/plants12051192/s1, Supplementary Table S1 and Supplementary Data File.
